# On Essential Oil of Ginger

**Published:** 1853-07

**Authors:** A. Papousek


*On Essential Oil of Ginger.* By A. Papousek.—The root of *Zingiber officinale* contains, according to Morin, an essential oil. To obtain this in sufficient quantity, ginger was submitted to distillation with water. With the water a yellow oil goes over, which possesses in a high degree the odor of ginger, and a burning aromatic taste. Its boiling-point is 475° F., its specific gravity 0.893.

The raw oil was deprived of water by pieces of fused chloride of calcium, and kept in a retort at a temperature below its boiling-point. At 302° F. a colorless oil evaporated, which, on analysis, gave the following numbers :—

| | | |
|---|---|---|
| Carbon . . . . . | 81.03 | 80=81.49 |
| Hydrogen . . . . . | 11.58 | 69=11.72 |
| Oxygen . . . . . | 7.39 | 5= 6.79 |

$C_{80} H_{69} O_5 = C_{80} H_{64} + 5HO$. This oil is therefore a mixture of hydrates of a hydro-carbon isomerous with oil of turpentine.

As the oil acquired a darker color, and began to undergo decomposition (as was known by the formation of water) when the heat was continued, the distillation was carried no further.

The raw oil was repeatedly distilled with anhydrous phosphoric acid. The yellow distillate gave the following numbers on analysis :—

| | | |
|---|---|---|
| Carbon . . . . . | 87.99 | 10=88.24 |
| Hydrogen . . . . | 11.88 | 8=11.76 |

The formula $C_{10} H_8$ places this oil with the numerous series of hydocarbons usually characterized as the camphene series. The separation of the hydrate-water appears to be effected with equal ease by the action of muriatic acid, as by that of anhydrous phosphoric acid.

If muriatic acid gas be passed into the raw oil, the latter acquires a brown color, even if care has been taken by cooling that the action should not be too violent. The brown oil, saturated with muriatic acid, was washed with water, then submitted to distillation with water, and the product, which is of a yellowish color, and contains chlorine, dried over chloride of calcium. As shown by analysis, these operations, employed for the purpose of purification, partially decompose the muriatic acid compound, forming a mixture of a muriatic acid compound in an unchanged state with a hydrocarbon which has lost its muriatic acid. Analysis gave—

| | | | |
|---|---|---|---|
| Carbon . . . . . | 73.39 | 80 | 73.45 |
| Hydrogen . . . . . | 10.36 | 67 | 10.25 |
| Chlorine . . . . . . . . | | 3 | 16.30 |

$C_{80} H_{67} Cl_3 = C_{80} H_{64} + 3Cl H$. This formula may be split in the following manner :—$3 (C_{20} H_{16}, ClH) + C_{20} H_{16}$.

According to this, the essential oil of ginger belongs to the same class of essential oils as the coriander oil. The ginger employed so plentifully as an aromatic in cookery, must therefore also belong to the camphene group of aromatics.—*Lond. Pharm. Journal, from Sitzungsb. der Akad der Wissench. zu Wien, and Chem. Gazette.*

